# Editorial: Perspectives in neuroscience: mechanical forces for the modulation of axonal mechanics and nerve regeneration

**DOI:** 10.3389/fnmol.2024.1453190

**Published:** 2024-07-12

**Authors:** Vittoria Raffa, Orit Shefi, Alessandro Falconieri

**Affiliations:** ^1^Department of Biology, Università di Pisa, Pisa, Italy; ^2^Faculty of Engineering, Bar Ilan Institute of Nanotechnologies and Advanced Materials, Gonda Brain Research Center, Bar Ilan University, Ramat Gan, Israel; ^3^Division of Neuroscience, Department of Brain Sciences, Imperial College London, London, United Kingdom

**Keywords:** force, axon, nerve regeneration, mechanotransduction, cytoskeleton

Nerve injury is a pervasive clinical issue with profound social and economic implications worldwide. Despite the availability of different therapeutic schemes for stimulating nerve regeneration, these treatments have not yielded significant benefits for patients, especially for injuries of the central nervous system (CNS). Consequently, it is essential to return to basic research to explore new avenues and develop more effective solutions for nerve repair and regeneration. This has encouraged scientific and public interest in the research aimed at understanding and promoting axon regeneration post-injury. Historically, chemical signaling in axon growth has long been recognized, but recent attention has shifted toward the role of the biophysical environment and axonal mechanics. Mechanical force plays a key role in the morphogenesis of the CNS, modulating neural cell (neurons and precursors) navigation and pathfinding. There is growing interest in understanding whether the molecular mechanisms underlying this process can be harnessed to develop new approaches for nerve regeneration. Mechanical forces, including tension and compression, are now recognized for their critical role in modulating axonal outgrowth and repair. This Research Topic aims to pave a stimulating discourse in the field. Here, we present three original articles and one review article that offer new insights, pushing the boundaries of our current understanding and contributing to the ongoing dialogue in the field. In the last decades, the field of neuronal mechanics experiences a renaissance due to the application of new methodologies and tools that can accurately and non-invasively stimulate axons or study the intrinsic mechanisms.

In Chouhan et al. and Falconieri et al. two different methods for investigating the effects of mechanical forces in neurons have been presented. Chouhan et al. developed micro-tissue engineered neural networks (micro-TENNs) (Cullen et al., 2012) in which a hydrogel micro-column structure enables the elongation of axon tracts in a 3D geometry that recapitulates aspects of axon fasciculation. The data suggest that bundled axons have capacity to generate contractile mechanical forces following target integration. These contractile forces can, in turn, generate tensile forces to induce “stretch-growth” (i.e. axon growth mediated by stretching) and microtubules seems to be responsible for axonal contractility.

Interestingly, microtubule stabilization is also described as the key factor for stretch-growth in the paper of Falconieri et al.. Here the authors use nano-pulling for stretching axons of dorsal root ganglia (DRG), a method consisting in loading axons with magnetic nanoparticles and generating a traction force through magnetic fields (Falconieri et al., 2023). Briefly, nano-pulling-induced microtubule stabilization led to an increase in axonal mass, activation of local translation and increased neuronal maturation in a tissue model of axotomy consisting of transected DRG, proving insights for the future application of nano-pulling as a therapy for injured neurons.

Microtubule stabilization seems to play a pivotal role in the mechanisms governing axonal responses to different type of forces. The review article by Coppini et al., further explores this Research Topic. Specifically, this work focuses on the study of mechanical fatigue, understood as the failure of a material after repeated application of a stress, eventually leading to failure at loads far below the yield stress of the material (Suresh, 1998). The review then focuses on mechanical stress experienced by neurons, distinguishing between extension/stretching forces, compression and bending/shear stress. Here, the role of the cytoskeleton is in-depth evaluated and recognized as a key mechanism of the cellular response to mechanical stress. Emphasis is placed on the challenge of imaging these cytoskeletal dynamics to study fatigue and mechanical degradation, and the prospect of new microscopy techniques. Understanding repair mechanisms and long-term stability is an emerging topic with implications at the intersection of biology and engineering and may inspire new therapeutic approaches.

However, microtubules are not the only component of the cytoskeleton involved. Qiu et al., investigated the role of actin cytoskeleton in mediating the cross-talk between chemical and mechanical signaling. An emerging and fascinating hypothesis is that mechanical force is a downstream effector of many chemical guidance cues. Here, the authors focus on Netrin-1 signaling. Netrin-1 is a chemical cue that guides axon growth by interacting with its receptor DCC, which connects to the actin cytoskeleton via the clutch-linker molecule shootin1a (Toriyama et al., 2013). Specifically, they demonstrated the interaction between DCC and shootin1a and the ability of this interaction to couple F-actin flow with substrate-bound Netrin-1, verifying that the force generated by shootin1a-mediated actin-DCC coupling is a key requirement for Netrin-1-induced axon growth and haptotaxis.

In summary, these studies presented in this Research Topic demonstrate the potential of mechanobiology in neuroscience, suggesting that importance of understanding cytoskeleton dynamics and the chemical-mechanical cross-talk: the future of nerve repair may lie not only in the understanding of chemical signaling but also in the biophysics of neural tissues and in the integration between these two stimuli.

## Author contributions

VR: Conceptualization, Funding acquisition, Project administration, Writing – review & editing. OS: Conceptualization, Funding acquisition, Project administration, Writing – review & editing. AF: Conceptualization, Funding acquisition, Project administration, Writing – original draft, Writing – review & editing.
